# New oligonucleotide microarray for rapid diagnosis of avian viral diseases

**DOI:** 10.1186/s12985-017-0738-0

**Published:** 2017-04-05

**Authors:** Kulyaisan T. Sultankulova, Nurlan S. Kozhabergenov, Vitaliy M. Strochkov, Yerbol D. Burashev, Kamshat A. Shorayeva, Olga V. Chervyakova, Nurkuisa M. Rametov, Nurlan T. Sandybayev, Abylay R. Sansyzbay, Mukhit B. Orynbayev

**Affiliations:** Research Institute for Biological Safety Problems (RIBSP), Science Committee of RK ME&S, Gvardeiskiy, 080409 Republic of Kazakhstan

**Keywords:** Avian infections, Virus, Rapid diagnosis, Microarray, Probe

## Abstract

**Background:**

We developed a new oligonucleotide microarray comprising 16 identical subarrays for simultaneous rapid detection of avian viruses: avian influenza virus (AIV), Newcastle disease virus (NDV), infection bronchitis virus (IBV), and infectious bursal disease virus (IBDV) in single- and mixed-virus infections. The objective of the study was to develop an oligonucleotide microarray for rapid diagnosis of avian diseases that would be used in the course of mass analysis for routine epidemiological surveillance owing to its ability to test one specimen for several infections.

**Methods and results:**

The paper describes the technique for rapid and simultaneous diagnosis of avian diseases such as avian influenza, Newcastle disease, infectious bronchitis and infectious bursal disease with use of oligonucleotide microarray, conditions for hybridization of fluorescent-labelled viral cDNA on the microarray and its specificity tested with use of AIV, NDV, IBV, IBDV strains as well as biomaterials from poultry.

Sensitivity and specificity of the developed microarray was evaluated with use of 122 specimens of biological material: 44 cloacal swabs from sick birds and 78 tissue specimens from dead wild and domestic birds, as well as with use of 15 AIV, NDV, IBV and IBDV strains, different in their origin, epidemiological and biological characteristics (RIBSP Microbial Collection). This microarray demonstrates high diagnostic sensitivity (99.16% within 95% CI limits 97.36–100%) and specificity (100%). Specificity of the developed technique was confirmed by direct sequencing of NP and M (AIV), VP2 (IBDV), S1 (IBV), NP (NDV) gene fragments.

**Conclusion:**

Diagnostic effectiveness of the developed DNA microarray is 99.18% and therefore it can be used in mass survey for specific detection of AIV, NDV, IBV and IBDV circulating in the region in the course of epidemiological surveillance. Rather simple method for rapid diagnosis of avian viral diseases that several times shortens duration of assay versus classical diagnostic methods is proposed.

## Background

Intensive poultry farming leads to higher risk of infectious disease emergence causing great economical losses. Boundary spanning between clinical manifestations of different agents is peculiar to the course of many infections nowadays. More and more infectious diseases progress in association with different microorganisms and it effects significantly the clinical manifestation and differential diagnosis of the disease.

Currently, the viral infections such as avian influenza, Newcastle disease, infectious bronchitis, and infectious bursal disease, etc., are a potential threat to poultry farming in the Republic of Kazakhstan. Monitoring these economically significant avian diseases is the question of the day for poultry industry.

Avian influenza virus belongs to the Orthomyxoviridae family, Influenza A virus genus. From the beginning of year 2016 the disease outbreaks were recorded in 30 countries [1]. Different AIV strains can cause 10 to 100% mortality among poultry.

The agent of the Newcastle disease is an RNA-containing virus, a member of the Paramyxoviridae family, Rubulavirus genus. In 2016 13 countries reported Newcastle disease cases to the OIE [1]. In poultry industrial farms, all infected birds need to be sacrificed due to threat of dissemination of the infection across countries [2].

The agent of the infectious bursal disease is RNA-containing virus of Avibirnavirus genus in Birnaviridae family. In outbreaks of the infectious bursal disease practically the entire population is affected and the lethality rate can approach 90% [3], the reconvalescent birds become susceptible to the majority of infectious diseases of viral and bacterial etiology [4].

The causative agent of infectious bronchitis is an RNA-containing Coronavirus avia of Coronavirus genus in Coronaviridae family [5]. Economical losses due to infectious bronchitis is composed of reduced egg and meat productivity, compulsory slaughter of sick birds, high death rate in young population. When the infection circulates in the farm for the first time the lethality rate can reach 70% [6].

Currently, standard immunological methods [7] or methods based on polymerase chain reaction (PCR) [8, 9] are widely used to identify the above mentioned viruses. Unfortunately, they can detect only one agent in a specimen.

There are also multiplex RT-PCR assays that make possible simultaneous detection of more than one infectious agent by using multiple primer pairs. Advantage of the multiplex RT-PCR is in combination of sensitivity and quickness of PCR alongside with elimination of need to test clinical specimens for each agent separately [10, 11].

Avian viruses can cause diseases independently, in alliance with each other or in association with bacterial agents [12]. Thereby, rapid and sensitive methods of detection are required that are able to differentiate viral infections for surveillance of newly emerging avian viruses as well as for disease control.

Application of DNA microarray technology that makes possible multivariate analysis of genetic material is a highly promising way for simultaneous detection of several agents (AIV, NDV, IBV and IBDV) in one specimen.

The paper describes the technique for rapid and simultaneous diagnosis of avian diseases such as avian influenza, Newcastle disease, infectious bronchitis and infectious bursal disease with use of oligonucleotide microarray, conditions for hybridization of fluorescent-labelled viral cDNA on the microarray and its specificity tested with use of AIV, NDV, IBV, IBDV strains as well as biomaterials from poultry.

The objective of this study is to develop an oligonucleotide microarray for rapid diagnosis of avian influenza, Newcastle disease, infectious bronchitis, and infectious bursal disease that will be used in the course of mass analysis for routine epidemiological surveillance owing to its ability to test one specimen for several infections.

## Methods

### Virus strains and clinical materials

Four AIV and 4 NDV strains, 2 IBV and 5 IBDV strains from the RIBSP (ME&S RK/SC) microbial collection were used in the study (see Table 1 for the list of these strains).

**Table 1:** Viruses from the RIBSP microbial collection that were used in the work

№	Strain	Taxonomy of virus	Strain description	Year of isolation
Avian influenza virus				
1	“A/duck/Alberta/35/76” (H1N1)	Genus-*Influenza virus A*, Family-*Orthomyxoviridae*	virulent	2015
2	“A/duck/Germany/215” (H2N3)		virulent	2013
3	“A/duck/California/72” (H3N8)		virulent	2013
4	“A/duck/Czechoslovakia/56” (H4N6)		virulent	2013
Newcastle disease virus				
5	“Columba livia /KZ/EKO/15/2014”	Genus-*Rubulavirus,* Family-*Paramyxoviridae*	virulent	2014
6	“Bor-74 VGNKI “		vaccinal	2015
7	“52/98”		virulent	2002
8	“63/00”		virulent	2002
Infectious bursal disease virus				
9	“Vinterfild”	Genus-*Avibirnavirus,* Family-*Birnaviridae*	vaccinal	2004
10	“201”		virulent	2003
11	“Koktal”		virulent	1998
12	“52–70”		virulent	1997
13	“BG”		vaccinal	2004
Infectious bronchitis virus				
14	“H-120”	Genus-*Coronavirus,* Family-*Coronaviridae*	vaccinal	2015
15	“10–95”		virulent	2005

**Table 2:** Description of samples from wild and domestic birds on the territory of Kazakhstan

Family	Bird species	Place of isolation	Sample number, *n* = 122	Cloacal swabs, *n* = 44	Tisssue samples, *n* = 78
*Corvidae*	Rook (*Corvus frugilegus)*	Ornithological station “Shakpak”, Zhambyl region	6	3	3
*Fringillidae*	Bramble finch *(Fringilla montifringilla)*	Ornithological station “Shakpak”, Zhambyl region	8	4	4
*Sturnidae*	Starling *(Sturnus vulgaris)*	Ornithological station “Shakpak”, Zhambyl region	3	2	1
*Anatidae*	Wild duck *(Anas platyrhynchos)*	Tasytkol Lake, Zhambyl region	10	6	4
*Phalacrocoracidae*	Great cormorant *(Phalacrocorax carbo)*	Alakol Lake, Almaty Region	10	3	7
*Pelecanidae*	Dalmatian pelican *(Pelecanus crispus)*	Alakol Lake, Almaty Region	4	2	2
*Galliformes*	Broiler chickens *(Gallus gallus)*	Poultry factory “Allel Agro”, Almaty Region	46	14	32
*Galliformes*	Domestic chickens *(Gallus gallus domesticus)*	Korday district, Zhambyl region	35	10	25

One-hundred and twenty-two samples (44 cloacal swabs from sick birds and 78 tissue samples from dead ones) were delivered by veterinarians from different regions of Kazakhstan to RGE RIBSP in the routine epidemiological surveillance for diagnosing sickness and death of birds (Table 2).

### RNA extraction

RNA was extracted from virus-containing material with TRizol (“Invitrogen”, USA) according to the manufacturer's instruction.

### Selection of oligonucleotide primers and probes

For selection of oligonucleotide primers and probes as microarray components the representative sample from International Data Base of NCBI (National Center for Biotechnological Information) GenBank (https://www.ncbi.nlm.nih.gov/) containing genomes of AIV, NDV, IBV and IBDV was used. All full-sized encoding sequences of virus nucleotides were aligned by using Clustal W algorithm in Mega 6.0 software by method of progressive multiplex alignment. Oligonucleotide probes with optimal physicochemical characteristics were selected with use of OligoWiz 2.1 and Picky 2.20 programs.

Specificity of the chosen oligonucleotide primers and probes was tested with use of BLAST (Basic Local Alignment Search Tool) program that could compare the existing sequence with sequences in database in NCBI BLAST-analysis (NCBI, https://blast.ncbi.nlm.nih.gov/Blast.cgi).

Oligonucleotide primers and probes were synthesized in DNA/RNA Synthesizer H-16 (K&A Laborgeraete, Germany) according to the manufacturer's instruction.

### PCR amplification

PCR-amplification was carried out in multi-prime format. RT-PCR was performed with use of Super Script III One-Step RT-PCR System with Platinum Taq (Invitrogen, USA) according to the manufacturer's instruction (in 50 μl of reaction mixture: Super Script III RT/Platinum Taq Mix-1 μl; 2x Reaction Mix-up to 1x; primers 20 pmol-1 μl each; RNA-5 μl each; DEPC-treated water up to 50 μl). Mixture of primers complementary to NP and M (AIV), VP2 (IBDV), S1 (IBV), NP (NDV) gene regions was used for specimen amplification. Fluorescent labeling of specimens was carried out by direct embedding of Cy5-dCTP (“Amersham”, USA) in the process of RT-PCR, the reaction mixture was supplemented in this case with 2 μl of 1 mM Cy5-dCTP.

### Microarray printing

Oligonucleotide probes were diluted 1:1 with twofold buffer for oligonucleotide printing (“Arrayit corporation”, USA), in concentration 50 pmol applied on glass slides without support (“Sigma”, USA) by a method of contact printing with use of NanoPrint LM60 spot-printer (“Arrayit corporation”, USA). Slide contained 16 arrays, where oligonucleotide probes complementary to certain AIV, NDV, IBV, IBDV genome loci were identically immobilized as separate spots 300 μm in diameter.

### Hybridization

To 1.0 μl of PCR-mixture containing Cy5-cDNA hybridization solution was added, the total volume was brought with H_2_O up to 50.0 μl and heated in the solid-state thermostat at 99 °C for 2 min, then cooled in ice for 2 min and at once applied onto the microarray. In parallel the oligonucleotide probes on the microarray were denatured by boiling of the slide in H_2_O for 1 min followed by incubation in 96% ethanol (−20 °C) for 1 min. After that the slide was dried by centrifugation at 300 g for 2 min. Hybridization was performed with use of a frame for 16 subarrays FAST® Frame (“Whatman”, USA) for 2 h at 37 °C and stirring at 250 rpm. After hybridization the slide was rinsed in 3 × SSC buffer for 2 min and in 1 × SSC buffer for 2 min to remove unbound molecules of the sample and hybridization buffer. After that the frame was removed and the slide was rinsed with water for 2 min. It was dried by centrifugation at 300 g for 2 min.

### Microarray scanning

Microarray scanning was carried out by use of InnoScan710AL scanner (“Innopsys”, France) at 5 μm resolution and wavelengths 532 nm and 633 nm. The resulted data were processed with the help of Mapix ver. 5.5.0 software (“Innopsys”, France) and the matrix corresponding to the probe layout on microarray was applied on the obtained pixel image. Afterwards the applied array was used to detect probes by intensity of fluorescence with quantitative output with the help of the program module. Median fluorescence values of probes minus background signals were considered as effective data.

### Nucleotide sequencing

The BigDye Terminator Cycle Sequencing kit was used according to the manufacturer’s instructions. The sequencing run was carried out using the 16-capillary ABI PRISM 3130 xl Genetic Analyser, (Applied Biosystems).

### Specificity and accuracy of the oligonucleotide microarray

The specificity of the assay was theoretically assessed by evaluating the primers and probes for relevant homologies using the BLAST tool (https://blast.ncbi.nlm.nih.gov/Blast.cgi).

### Real-time RT-PCR

Real-time RT-PCR was performed with use of Light Cycler 2.0 manufactured by Roche Company to detect AIV [13], NDV [14], IBV [15] and IBDV [16].

### Statistical treatment

In assessment of laboratory tests effectiveness truepositive (TP), truenegative (TN), falsepositive (FP) and falsenegative (FN) results of the assays were used.

The following calculations were used: Sensitivity (SN) = (TP/TP + FN), Specificity (SP) = (TN/TN + FP), Positive Predictive Value PPV = (TP/TP + FP), Negative Predictive Value (NPV) = (TN/TN + FN), diagnostic effectiveness (DE) = (TP + TN/TP + FP + FN + TN) [17]. Ninety-five percent confidence intervals (95% CI) were calculated according to Wilson [17].

## Results

### Oligonucleotide microarray for rapid detection of AIV, NDV, IBV, IBDV

In the result of analysis of nucleotide sequences from NCBI “Influenza Virus Sequence Database“genes encoding M and NP proteins were chosen for AIV identification. Nucleotide sequences of gene encoding NP protein were selected for NDV. Segment A of VP2 protein was used for IBDV. Nucleotide sequences of gene encoding S1 protein were chosen for IBV identification.

Oligonucleotide primers and probes were produced in the course of standard automatic synthesis in DNA/RNA Synthesizer H-16 (K&A Laborgeraete, Germany), their sequences are shown in Tables 3 and 4.

**Table 3:** Oligonucleotide primers for detection of AIV, NDV, IBV, and IBDV

Name	Primer sequence (5` → 3`)	Position in genome	PCR product size, bp
IBDV-VP2	(F) GAGCTGATCCCAAATCCTGAA (R) GCGTCTTCCACTGTCGTAATAA	1244–1264 1836–1815	593
IBV-S1	(F) TATGGCAGAACTGGCCAAGG (R) AAGGTGCCACAAACTGTTCC	21,622–21,641 22,042–22,023	421
NDV-NP	(F) ATGACATTGCTAGGCGACAG (R) GAATTGTGTCTCTCCGTCCC	1051–1070 1461–1442	411
AIV Flu-NP	(F) GGAACCACCAACCAACAGA (R) TCCTCTGCATTGTCTCCGAA	1180–1198 1484–1465	305
AIV Flu-M	(F) TCTCATAGGCAAATGGTGGC (R) AGACTCAGGTACTCCTTCCG	506–525 926–907	421

**Table 4:** Oligonucleotide probes that were used in the microarray for diagnosis of avian infections

Probes	Oligonucleotides sequence (5` → 3`)	Length, nucleotides	Virus	Position in genome	Gene
IBDV-VP2	AGGCACAGGCTGCTTCAGGAACTGCTCGAGCCGCGTCAGGAAAAGCAAGRGCTGCCTCAGGCCGCA	66	IBDV	1581–1640	VP2 (segment A)
IBV-S1	TTTCTGGTGGTAAATTAGTAGGTATTCTYACTTCACGTAATG	42	IBV	21,812–21,853	S1
AIV Flu-NP	ACGAAAAGGCAACGAACCCGATCGTGCCTTCCTTTGACATGA	42	AIV	1403–1444	NP
AIV Flu-M2	CCTATCAGAAACGAATGGGGGTGCAGATGCAACGATTCAAGTGA	44	AIV	716–759	M2
NDV-NP	AACAGGCCGGGGTCCTCACTGGGCTCAGCGACGAAGGTCCCCGAGCCC	48	NDV	1353–1400	NP

The selected oligonucleotide probes were used to develop a microarray for rapid diagnosis of AIV, NDV, IBV, and IBDV. Probes were applied on the support by the method of contact printing in Nano Print LM60 (Arrayit Corp., USA).

**Fig. 1:**
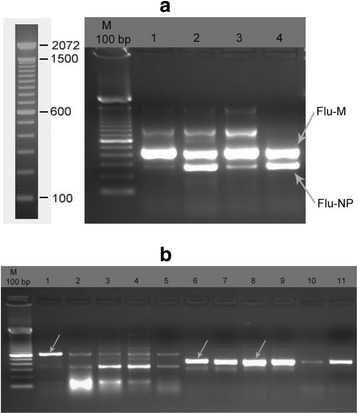
PCR products of NP and M (AIV), VP2 (IBDV), S1 (IBV), NP (NDV) gene fragments. **a**) M – 100 bp marker (Invitrogen), 1 – “A/duck/Alberta/35/76” (H1N1) (AIV); 2 – “A/duck/Germany/215” (H2N3) (AIV); 3 – “A/duck/California/72” (H3N8) (AIV); 4 – “A/duck/Czechoslovakia/56” (H4N6) (AIV). **b**) M – 100 bp marker (Invitrogen), 1 – “Vinterfild” (IBDV); 2 – “BG” (IBDV); 3 – “201” (IBDV); 4 – “Koktal” (IBDV); 5 – “52/70” (IBDV); 6 – “H-120” (IBV); 7 – “10-95” (IBV); 8 – “63/00” (NDV); 9 – “52/98” (NDV); 10 – “Bor-74 VGNKI” (NDV); 11 – “Columba livia/KZ/EKO/15/2014” (NDV)

The study has shown that amplification products hybridize on the microarray only in case of obtaining sufficient number of single-chain fluorescently-labeled fragments of NP and M (AIV), NP (NDV), VP2 (IBDV), and S1 (IBV). The electrophoregram has shown PCR products-305 bp (AIV), 411 bp (NDV), 421 bp (IBV), 593 bp (IBDV) (Fig. 1).

**Fig. 2:**
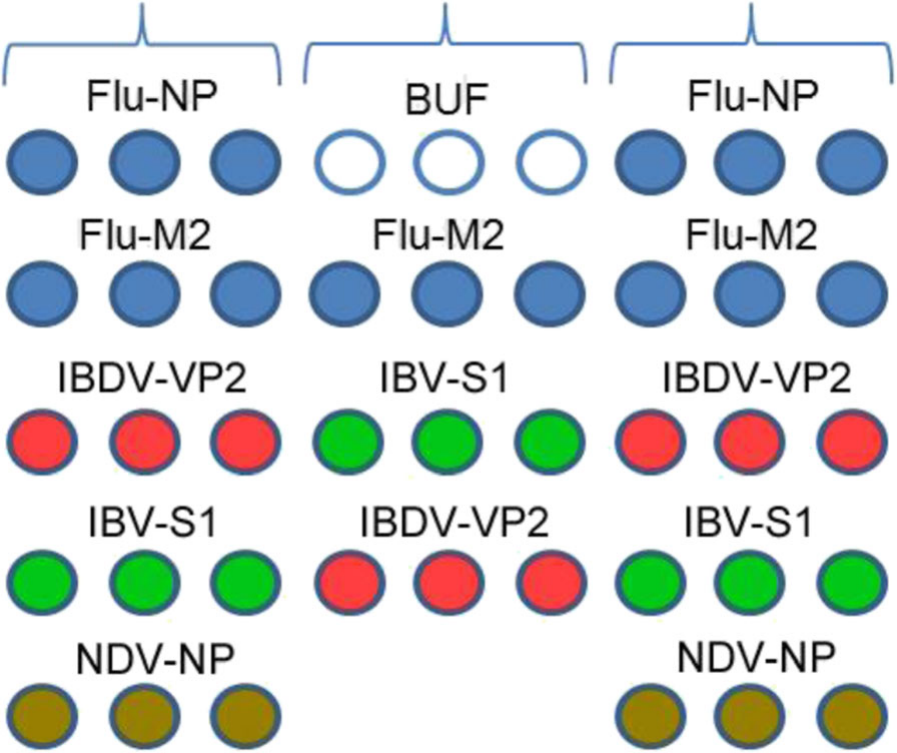
Layout of oligonucleotide probes on one array of DNA microarray for detection of avian influenza virus (*Flu-NP*, *Flu-M2*), infectious bursal disease virus (*IBDV-VP2*), infectious bronchitis (*IBV-S1*) and Newcastle disease virus (*NDV-NP*). BUF - Micro spotting solution Plus (Arrayit, USA)

Hybridization on the microarray of the obtained fluorescently-labeled fragments of NP and M2 (AIV), VP2 (IBDV), S1 (IBV), NP (NDV) genes of the viruses under study resulted in formation on glass slide of stable hybridization complexes with high binding energy and oligonucleotides the sequences of which were complementary to sequences of hybridized NP and M2 of AIV, VP2 of IBDV, S1 of IBV, NP of NDV gene fragments. For interpretation of the results the microarray layout is shown on Fig. 2. The first two horizontal rows contain universal oligonucleotide probes to NP and M2 genes of AIV, next are probes for detection of VP2 gene of IBDV and S1 gene of IBV. Last row contains probes for detection of NP gene of NDV.

In the microarray layout there are 16 identical subarrays arranged into 2 columns and 8 rows containing oligonucleotide probes that are complementary to antisense strand of AIV, NDV, IBV and IBDV genes.

**Fig. 3:**
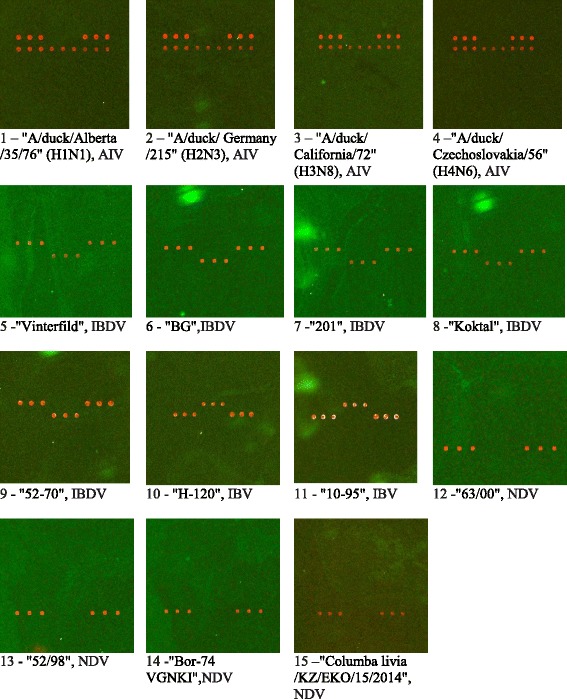
Results of scanning labeled cDNAs of AIV, NDV, IBV and IBDV strains on microarray. 1 – "A/duck/Alberta/35/76" (H1N1) (AIV); 2 – "A/duck/Germany/215" (H2N3) (AIV); 3 – "A/duck/California/72" (H3N8) (AIV); 4 – "A/duck/Czechoslovakia/56" (H4N6) (AIV); 5 – "Vinterfild" (IBDV); 6 – "BG" (IBDV); 7 – "201" (IBDV); 8 – "Koktal" (IBDV); 9 – "52/70" (IBDV); 10 – "H-120" (IBV); 11 – "10-95" (IBV); 12 – "63/00" (NDV); 13 – "52/98" (NDV); 14 – "Bor-74 VGNKI" (NDV); 15 – "Columba livia/KZ/EKO/15/2014" (NDV)

**Fig. 4:**
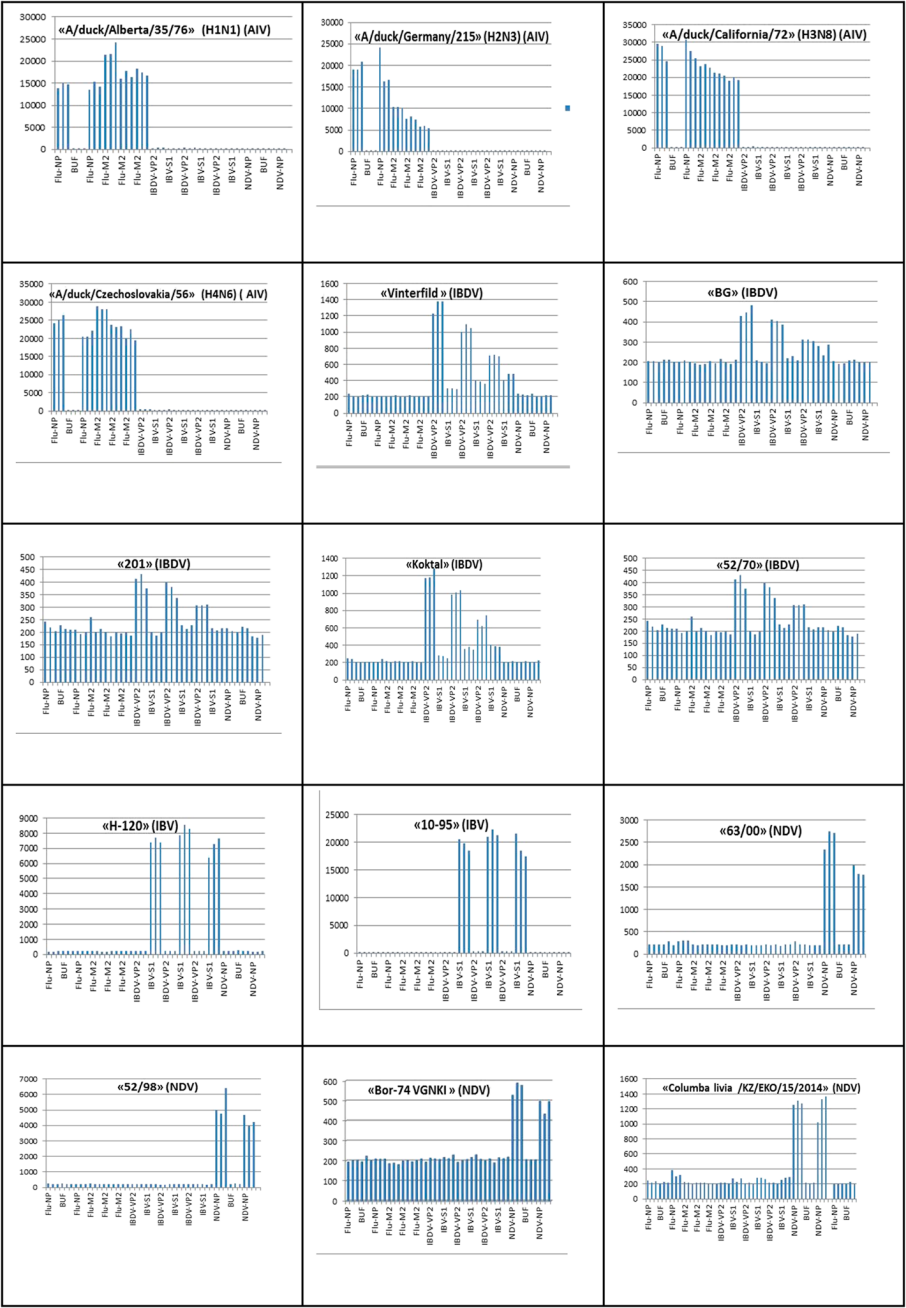
Intensity profiles of hybridization signals of labeled cDNAs of strains "A/duck/Alberta/35/76" (H1N1) (AIV); "A/duck/Germany/215" (H2N3) (AIV); "A/duck/California/72" (H3N8) (AIV); "A/duck/Czechoslovakia/56" (H4N6) (AIV); "Vinterfild"(IBDV); "BG" (IBDV); "201" (IBDV); "Koktal" (IBDV); "52-70" (IBDV); "H-120" (IBV); "10-95" (IBV); "63/00" (NDV); "52/98" (NDV); "Bor-74 VGNKI" (NDV); "Columba livia /KZ/EKO/15/2014"(NDV)

The DNA-chip was scanned with InnoScan710AL (“Innopsys”, France) by Cy5 channel activation. The results were interpreted with use of Mapix ver. 5.5.0 software. The signal exceeding the background value was adopted as a positive result. The finding of the study considered reliable if in the course of scanning by Cy5 channel bright fluorescent spots were observed. In the assay of samples the value of specific fluorescence reliably exceeded the value of the background signal (*P* < 0.05) (Figs. 3 and 4).

As Figs. 3 and 4 show M2 and NP genes of AIV, VP2 gene of IBDV, S1 gene of IBV and NP gene of NDV were reliably detected in all specimens.

For testing specificity of the method direct sequencing of the PCR-products in ABI PRISM 3130 xl Genetic Analyser, Applied Biosystems was carried out. The synthesized nucleotide sequences were analyzed using BLAST program. These nucleotide sequences that are the fragments of NP and M2 (AIV), VP2 (IBDV), S1 (IBV), NP (NDV) genes were compared with the data of the Genbank Database. Computer analysis has shown the amplified specific PCR-products of AIV, NDV, IBV, IBDV to be parts of their genomes. So, homology of compared sequences confirms specificity of the developed method. The results of the performed experiments prove again that the used fragments of tested genes are highly specific for AIV, NDV, IBV, IBDV. The limit of detection of the assay method is 10^2^ copies of RNA.

### Testing of the microarray with use of viruses from the RIBSP microbial collection

Different strains of AIV, NDV, IBV and IBDV were used to test the oligonucleotide microarray. Testing was carried out in comparison with real-time RT-PCR (Table 5).

**Table 5:** Detection of AIV, NDV, IBV and IBDV strains with use of real-time RT-PCR and microarray

№	Virus	Real-time RT-PCR				Microarray			
		AIV	NDV	IBV	IBDV	AIV	NDV	IBV	IBDV
1	“A/duck/Alberta/35/76” (H1N1) (AIV)	+++	− − −	− − −	− − −	+++	− − −	− − −	− − −
2	“A/duck/Germany/215” (H2N3) (AIV)	+++	− − −	− − −	− − −	+++	− − −	− − −	− − −
3	“A/duck/California/72” (H3N8) (AIV)	+++	− − −	− − −	− − −	+++	− − −	− − −	− − −
4	“A/duck/Czechoslovakia/56” (H4N6) (AIV)	+++	− − −	− − −	− − −	+++	− − −	− − −	− − −
5	“Vinterfild “(IBDV)	− − −	− − −	-	+++	− − −	− − −	-	+++
6	“BG” (IBDV)	− − −	− − −	-	+++	− − −	− − −	-	+++
7	“201” (IBDV)	− − −	− − −	-	+++	− − −	− − −	-	+++
8	“Koktal” (IBDV)	− − −	− − −	-	+++	− − −	− − −	-	+++
9	“52/70” (IBDV)	− − −	− − −	-	+++	− − −	− − −	-	+++
10	“H-120” (IBV)	− − −	− − −	+++	− − −	− − −	− − −	+++	− − −
11	“10-95” (IBV)	− − −	− − −	+++	− − −	− − −	− − −	+++	− − −
12	“63/00” (NDV	− − −	+++	− − −	− − −	− − −	+++	− − −	− − −
13	“52/98” (NDV)	− − −	+++	− − −	− − −	− − −	+++	− − −	− − −
14	“Bor-74 VGNKI “(NDV)	− − −	+++	− − −	− − −	− − −	+++	− − −	− − −
15	“Columba livia /KZ/EKO/15/2014” (NDV)	− − −	+++	− − −	− − −	− − −	+++	− − −	− − −

+++ virus is detected, *n* = 3

− − − virus is not detected, *n* = 3

Fifteen different strains of AIV, NDV, IBV and IBDV, diverse in their origin, epidemiological and biological characteristic, were identified correctly with use of DNA microarray. Diagnostic results of testing DNA microarray with use of virus strains from the RIBSP microbial collection were comparable to the results of the real-time RT-PCR. Sensitivity of the microarray was comparable to the sensitivity of real-time RT-PCR.

### Detection of viruses in field samples

In large-scale epidemiological studies possibility to analyze concurrently one specimen on many diagnostic probes is extremely important for agent identification. It allows minimizing the time of analysis from several days to several hours.

The microarray efficacy in rapid diagnosis of avian viral diseases was evaluated versus virus isolation in embryonated eggs and real-time qPCR for AIV, NDV, IBV and IBDV with use of 122 samples-44 cloacal swabs and 78 tissue samples from dead birds (Table 6).

**Table 6:** Virus detection in field samples by real-time RT-PCR, microarray and virus isolation in embryonated eggs

№	Virus	Real-time RT-PCR		Microarray		Virus isolation in embryonated eggs	
		Cloacal swabs	Tissue specimens from dead birds	Cloacal swabs	Tissue specimens from dead birds	Cloacal swabs	Tissue specimens from dead birds
1	AIV	7/44 (15.90%)	11/78 (14.10%)	7/44 (15.90%)	11/78 (14.10%)	7/44 (15.90%)	11/78 (14.10%)
2	NDV	28/44 (63.64%)	66/78 (84.62%)	28/44 (63.64%)	66/78 (84.62%)	28/44 (63.64%)	66/78 (84.62%)
3	IBV	0/44 (0%)	3/78 (3.85%)	0/44 (0%)	3/78 (3.85%)	0/44 (0%)	3/78 (3.85%)
4	IBDV	0/44 (0%)	2/78 (2.56%)	0/44 (0%)	3/78 (3.85%)	0/44 (0%)	3/78 (3.85%)
Positive		117 (95.90%)		118 (96.72%)		118 (96.72%)	

True disease status is determined by the most accurate diagnostic method possible that is called gold standard. In diagnosis of influenza and other avian infections it is virus isolation in chicken embryos followed by identification in hemagglutination inhibition test [18], the whole procedure taking from 2 to 5 days. In our study aimed at evaluation of the microarray and real-time RT-PCR effectiveness we used the test of virus isolation in chicken embryos as gold standard.

Among 44 cloacal swabs analyzed with use of microarray, real-time RT-PCR and virus isolation in embryonated eggs 15.90% of specimens were positive for AIV, 63.64% for NDV. None of cloacal swabs was shown to be IBV and IBDV positive.

AIV was detected by the microarray in 14.10% of 78 tissue samples from dead birds, NDV-in 84.62%, IBV-in 3.85% and IBDV-in 3.85% of cases. In real-time RT-PCR the picture is as follows: AIV-14.10%, NDV-84.62%, IBV-3.85% and IBDV-2.56%. In IBDV detection with use of real time RT-PCR 2.56% of samples appeared to be positive; in case of using the microarray and method of virus isolation in embryonated eggs the number of positives was 3.85%.

In 3 samples from dead domestic chickens (small households in Korday District of Zhambyl Region) the DNA microarray detected IBV (3.85%); IBDV was also detected in 3 samples (3.85%). Real Time PCR demonstrated IBV presence in 3 of the same samples from domestic chickens (3.85%) and IBDV presence in 2 samples (2.56%). Previously, the samples under study were shown to be NDV positive. The DNA microarray detected one case of NDV and IBV mixed infection and one case of NDV and IBDV mixed infection.

AIV was detected in wild birds: 66.67% samples from rooks (*Corvidae* family *Corvus frugilegus* species), 75.00% from bramble finches (*Fringillidae* family*, Fringilla montifringilla* species), 80.00% from wild ducks (*Anatidae* family, *Anas platyrhynchos* species), and not detected in samples from starlings *(Sturnidae* family*, Sturnus vulgaris* specie*s),* great cormorants *(Phalacrocoracidae* family*, Phalacrocorax carbo* species*),* Dalmatian pelican *(Pelecanidae* family*, Pelecanus crispus* species*),* as well as in samples from domestic chickens and chickens of the poultry factory.

NDV was detected in 90.00% of samples from great cormorants *(Phalacrocoracidae* family*),* 100% from Dalmatian pelican *(Pelecanidae* family) as well as in 100% of samples from broiler chickens of poultry factory “Allel Agro” (Almaty region) and in 100% of samples from dead domestic chickens in small households of Korday district (Zhambyl region). IBV and IBDV were detected respectively in 8.57 and 5.71% of samples from dead domestic chickens in small households of Korday district (Zhambyl region).

So, 118 samples of 122 were positive for avian infections being diagnosed with use of the DNA microarray and virus isolation in embryonated eggs, 117 samples displayed positive results in real-time RT-PCR. Diagnostic results of the DNA microarray testing with use of experimental specimens from sick and dead birds were comparable to the results of virus isolation in embryonated eggs and real-time RT-PCR.

Advantage of the DNA microarray is simultaneous assay of samples for presence of 4 infections-avian influenza, Newcastle disease, infectious bronchitis and infectious bursal disease of birds, while virus isolation in embryonated eggs and real-time RT-PCR allow detecting only one agent in a sample.

The results of AIV, NDV, IBV and IBDV detection in clinical specimens by different methods are shown in comparison with the results of virus isolation in embryonated eggs.

Sensitivity and specificity characteristics of the developed DNA microarray and of the real-time RT-PCR as well as positive and negative prognostic values at 95% confidence interval are shown in Table 7.

**Table 7:** AIV, NDV, IBV and IBDV detection in clinical specimens with use of different tests

Result	Real-time RT-PCR	Microarray	Virus isolation in chicken embryos
Positive	117 (95.90%)	118 (96.72%)	118 (96.72%)
Falsepositive	0	0	0
Falsenegative	1 (0.82%)	1 (0.82%)	1 (0.82%)
Negative	4 (3.29%)	3 (2.46%)	3 (2.46%)
Total	122 (100%)	122 (100%)	122 (100%)
SN, at 95% CI	99.15% (97.30–100%)	99.16% (97.36–100%)	Standard method for comparison
SP, at 95% CI	100%	100%	
PPV, at 95% CI	100%	100%	
NPV, at 95% CI	80.00% (71.96–88.04%)	75.00% (66.29–83.71%)	

*Abbreviations*: *SN* Sensitivity, *SP* Specificity, *PPV* Positive Predictive Value, *NPV* Negative Predictive Value, *95% CI* 95% confidence interval

While virus isolation in embryonated eggs was used as a standard method in our studies the DNA microarray demonstrated diagnostic sensitivity equal to 99.16% within 95% confidence limits 97.36–100%) and diagnostic specificity equal to 100%. High microarray sensitivity is comparable to the diagnostic sensitivity of the real-time RT-PCR equal to 99.15% within 95% CI limits 97.30–100%. Diagnostic specificity of the DNA microarray and real-time RT-PCR is 100%. Positive Predictive Values for the DNA microarray and real-time RT-PCR are 100%. Negative Predictive Values are 75 and 80% respectively.

## Discussion

Currently, most methods of AIV, NDV, IBV, IBDV and other avian viral agents detection are adapted to specific detection of one agent in a sample. Multiplex RT-PCR is successfully used for detection of AIV and its subtypes [19, 20] and for diagnosing double infections such as combination of NDV and AIV [21]. Also methods with use of multiplex real-time RT-PCR for AIV, NDV and IBV subtypes differentiation have been developed [22–24]. At present development of a test based on microarray technology for simultaneous detection of AIV, NDV, IBV and IBDV in one sample is important for poultry industry in the Republic of Kazakhstan.

Use of microarray improves quality and shortens the analysis duration in molecular diagnosis of infectious diseases and therefore is employed as an independent method in screening for several genes of large numbers of pathology samples [25–27]. There are biochips for influenza diagnosis that allow screening not only for HA and NA, but for M and NP genes of influenza A virus [25, 28]. In identification of NDV molecular methods with use of oligonucleotides specific to conservative regions of NP-gene of NDV were used [29]. Recently VP2 gene region of IBDV is successfully used in synthesis of oligonucleotide primers and probes from highly conservative regions for molecular diagnosis [30–33]. Molecular methods for IBV diagnosis are oriented at using more conservative sequences located in S1 and S2 genes of IBV [34, 35].

In the proposed microarray probes were developed on the basis of conservative regions of gene fragments encoding NP and M (AIV), NP (NDV), VP2 (IBDV), S1 (IBV) array proteins from Genbank Database. All viral gene fragments demonstrated high rate of conservatism and therefore the test is universal for detecting AIV, NDV, IBV and IBDV strains. So, high homology of nucleotide sequences of gene regions encoding AIV, NDV, IBV and IBDV array proteins compared to GenBank data confirms specificity of the developed microarray for rapid diagnosis of avian influenza, Newcastle disease, infectious bronchitis and infectious bursal disease.

Total analysis duration without time required for the viral RNA extraction is 5–6 h, and 16 specimens can be simultaneously assayed. Duration of the assay with use of the proposed microarray is not longer than in other molecular methods and simultaneous testing of samples for AIV, NDV, IBV and IBDV provides its advantage over other methods.

Various methods have been developed for the diagnosis of bird infection, such as virus isolation in cell culture, embryonated chicken eggs, or young specific-pathogen-free (SPF) chickens and localization of the virus in infected tissues by electron microscopy, fluorescence assay, agar immunodiffusion, antigene-capture enzyme-linked immunosorbent assay (ELISA), or immunohistochemistry. All these methods have disadvantages, such as being time consuming, labor intensive, expensive, or nonspecific. These methods lack the ability to detect low levels of antigens in tissues [36–40].

In the present study field samples (122 in total) were used to test effectiveness and reliability of the microarray. Nevertheless, positive result of using molecular and biological methods, being very important in emergency cases, should always be confirmed by the method of virus isolation.

The results of the study show that diagnostic sensitivity (99.16%) and diagnostic specificity (100%) of the DNA microarray are comparable with the same of the real-time RT-PCR (99.15 and 100%, respectively).

Diagnostic effectiveness as percentage ratio of true results to the total number of obtained results for the developed DNA microarray and real-time RT-PCR was 99.18%.

Analysis of the obtained data shows that the microarray test for rapid diagnosis of avian infections demonstrates the effectiveness comparable to that of the molecular method real-time RT-PCR and is more rapid and less resource-consuming owing to its ability to detect simultaneously AIV, NDV, IBV IBDV positive samples in the course of one experiment. Universality of the test makes it suitable for wide use in veterinary laboratories for prompt detection of avian infections.

## Conclusions

The developed microarray for rapid diagnosis of avian viral diseases can be used in mass analysis in the system of routine epidemiological surveillance owing to its ability to test one sample for simultaneous detection of AIV, NDV, IBV and IBDV in cases of single and mixed viral infections. At the same time duration of the analysis decreases many times versus classical methods and the proposed scheme of specimen preparation allows conducting assays immediately in small veterinary laboratories thus avoiding transportation of thermolabile RNA.

The study was conducted in years 2015–2016 under the grant research project (Ministry of Education and Science, Republic of Kazakhstan) “Development and testing of microarray for rapid diagnosis of avian viral diseases”, No. 0920/GF 4.

## Abbreviations

AIV: Avian influenza virus
IBDV: Infectious bursal disease virus
IBV: Infectious bronchitis virus
NDV: Newcastle disease virus
RT-PCR: Reverse-transcriptase polymerase chain reaction
